# Targeted human cytolytic fusion proteins at the cutting edge: harnessing the apoptosis-inducing properties of human enzymes for the selective elimination of tumor cells

**DOI:** 10.18632/oncotarget.26618

**Published:** 2019-01-25

**Authors:** Neelakshi Mungra, Sandra Jordaan, Precious Hlongwane, Krupa Naran, Shivan Chetty, Stefan Barth

**Affiliations:** ^1^ Medical Biotechnology and Immunotherapy Unit, Institute of Infectious Disease and Molecular Medicine, Faculty of Health Sciences, University of Cape Town, Cape Town 7700, South Africa; ^2^ South African Research Chair in Cancer Biotechnology, Department of Integrative Biomedical Sciences, Faculty of Health Sciences, University of Cape Town, Cape Town 7700, South Africa

**Keywords:** death-associated protein kinase (DAPk), granzyme B (GrB), targeted human cytolytic fusion proteins (hCFPs), microtubule-associated protein tau (MAPT tau), RNase-based hCFPs

## Abstract

Patient-specific targeted therapy represents the holy grail of anti-cancer therapeutics, allowing potent tumor depletion without detrimental off-target toxicities. Disease-specific monoclonal antibodies have been employed to bind to oncogenic cell-surface receptors, representing the earliest form of immunotherapy. Targeted drug delivery was first achieved by means of antibody-drug conjugates, which exploit the differential expression of tumor-associated antigens as a guiding mechanism for the specific delivery of chemically-conjugated chemotherapeutic agents to diseased target cells. Biotechnological advances have expanded the repertoire of immunology-based tumor-targeting strategies, also paving the way for the next intuitive step in targeted drug delivery: the construction of recombinant protein drugs consisting of an antibody-based targeting domain genetically fused with a cytotoxic peptide, known as an immunotoxin. However, the most potent protein toxins have typically been derived from bacterial or plant virulence factors and commonly feature both off-target toxicity and immunogenicity in human patients. Further refinement of immunotoxin technology thus led to the replacement of monoclonal antibodies with humanized antibody derivatives, including the substitution of non-human toxic peptides with human cytolytic proteins. Preclinically tested human cytolytic fusion proteins (hCFPs) have proven promising as non-immunogenic combinatory anti-cancer agents, however they still require further enhancement to achieve convincing candidacy as a single-mode therapeutic. To date, a portfolio of highly potent human toxins has been established; ranging from microtubule-associated protein tau (MAP tau), RNases, granzyme B (GrB) and death-associated protein kinase (DAPk). In this review, we discuss the most recent findings on the use of these apoptosis-inducing hCFPs for the treatment of various cancers.

## INTRODUCTION

### Conventional treatment approaches and their limitations

Despite the rise in scientific and technological progress, cancer remains a leading cause of death worldwide, accounting for 8.8 million deaths in 2015 alone (World Health Organization, 2018). For decades now, our primary lines of defense against cancer have involved surgery, chemotherapy and ionizing radiation. While many of these conventional therapies have offered substantial improvement in the survival of cancer patients, several shortcomings have been identified, which significantly outweigh their potential benefits. For instance, chemotherapy and radiation therapy are associated with various debilitating side-effects, arising primarily due to their lack of selectivity for malignant cells [1, 2]. Additionally, the overriding drawback associated with the use of cancer-killing drugs in chemotherapy, is the phenomenon of acquired or inherited drug resistance [3] in a small proportion of tumor cells, which may in turn promote aggressive tumor relapses. Similarly, although the removal of primary tumors by surgery may offer better survival chances, it is not effective in the face of metastasis, and is correlated to chronic pain, poor quality of life and psychosocial distress in patients [4, 5]. The limitations to mainstream treatments necessitates the need to explore novel solutions to tackle the ever-growing burden of disease.

### Targeted therapy, antibody-drug conjugates and the emergence of immunotoxins

Throughout history, the link between the etiology of cancer and its fundamental biological nature have been at the forefront of research [6]. Tumors can exhibit a completely different phenotype when exposed to drugs that have previously shown potential antitumor effect. Indeed, cancer is a multifactorial disease and using a ‘one-size-fits-all’ approach for its treatment is not adequate. With an increased understanding of the minutiae of the biochemical differences between normal and tumor cells, a more rational approach to drug design and patient-tailored therapies targeted to essential tumor-specific biochemical pathways was established, thereby shifting the focus from traditional chemotherapy to targeted cancer therapies. As speculated, with the development of molecular and genetic profiling platforms, innovative and promising tumor-directed cancer therapies have become more feasible; monoclonal antibodies, hormones and growth factors are now used to deliver drugs, toxins, photosensitizers and radionuclides to malignant cells [7].

The development of immunotherapy, a major breakthrough in medical science and oncology, was made possible through the use of bacterial toxins to force the immune system to induce a potent antitumor immune response [8]. Following this scientific milestone, introduction of a “magic-bullet” hypothesis paved the way for the expansion of antibody-based immunotherapy [9]. With the notion that cancerous cells express unique disease profiles, the use of antibodies could be instrumental in the selective delivery of toxic payloads to malignant cells. The advent of hybridoma technology, together with phage display techniques, fostered the generation of unlimited quantities of highly specific and diverse monoclonal antibodies (mAbs) [10, 11]. This suggests that antibodies with defined specificities could be produced to bind to most target antigens, thereby increasing the clinical potential of antibodies in cancer therapy.

The use of unconjugated mAbs as therapeutics has received considerable attention by the pharmaceutical industry; as of May 2018, approximately 80 mAbs have been approved by the Food and Drug Administration (FDA) for use in various indications, including cancer and immunological diseases [12, 13]. By selectively recognizing antigens that are preferentially expressed on tumor cells, mAbs can exert their cytotoxic effect through various mechanisms, including antibody-dependent cellular cytotoxicity, apoptosis, blocking growth factor receptors and complement-mediated cellular cytotoxicity [14]. Nonetheless, while the majority of these mAbs showed considerable utility in the treatment of cancer, they were rarely curative and were therefore subjected to various modifications [15]. Most importantly, mAbs were armed by conjugation with potent cytotoxic drugs, giving rise to antibody-drug conjugates (ADCs) [16].

Early work in ADC development involved the use of clinically approved and readily available drugs (such as doxorubicin, mitomycin and vinca alkaloids) and little attention was given to the mAb carrier, the mode and stoichiometry of drug attachment, and the mechanism of drug release [17]. As a result, access to solid tumors was dramatically hampered and accumulation of the drug in target cells was poor [18]. Additionally, multiple challenges became apparent: (1) only a limited number of drug molecules can be conjugated to the antibody without abrogating antigen binding, (2) producing homogeneous ADC populations, (3) the limited number of antigens on target cell surfaces can prevent therapeutic levels of drug accumulation in cells, and (4) ensuing serum stability [19]. Thus, improving the antibody, the linker and the toxic payload is essential to optimizing the functionality of ADCs.

An eloquent testament highlighting the difficulties involved in ADC development, is the result of approximately over 3 decades of research that has so far yielded only 3 clinically approved drugs: brentuximab vedotin, ado-trastuzumab emtasine (T-DM1) and inotuzumab ozogamicin [20]. In fact, the very first ADC to gain marketing approval by the US Food and Drug Administration (FDA) was gemtuzumab ozogamicin, consisting of a humanized anti-CD33 monoclonal antibody conjugated to the DNA-damaging agent calicheamicin, for the treatment of acute myeloid leukemia (AML) [21]. However, gemtuzumab ozogamicin was withdrawn from the market after subsequent clinical data raised concerns about the lack of safety and improved overall survival [20–22]. On the other hand, the introduction of antimitotic agents to the ADC development landscape has allowed expansion in the burgeoning ADC pipeline. For instance, two of the ADCs approved for cancer therapy, which employ microtubule-disrupting agents (auristatins and maytansines) as their payload, are brentuximab vedotin for the treatment of anaplastic large cell lymphoma (ALCL) and T-DM1 for use in HER2-positive breast cancer [23–25]. These compounds are able to kill cancer cells with IC_50_ values in the picomolar range, with a cytotoxicity of several orders of magnitude higher than clinically used anticancer agents such as doxorubicin and methotrexate [26]. Their exquisite cytotoxicity towards cancer cells as compared to slow-dividing cells, provide a buffer against adverse events in healthy tissues [27]. Similarly, the CD22-targeting inotuzumab ozogamicin, has shown improved complete remission and overall survival in adult acute lymphoblastic leukemia (ALL) [28, 29] and while the toxicities are manageable, some researchers raise the necessity for thorough preclinical evaluation of novel ADCs prior to advancement to the clinic [26].

While further optimization of ADC design continues to be an area of active research, the popular view that the immunotoxin approach has languished (as stated by Mullard (2013) [27]), is incorrect. With substantial progress in therapeutic protein deimmunization by the Pastan group, immunotoxins (ITs) such as moxetumomab pasudotox (composed of an anti-CD22 antibody genetically fused to PE38) and SS1P (an anti-mesothelin immunotoxin), are now showing durable complete responses and major tumor regressions in aggressive diseases such as relapsed/refractory hairy cell leukemia (HCL) and mesothelioma respectively [30–32].

ITs are highly potent molecules consisting of a cell-specific antibody covalently bound to a cytotoxic death-inducing effector component [33, 34]. Indeed, the 1^st^ and 2^nd^ generations of ITs consisted of native bacterial (*Pseudomonas aeruginosa* Exotoxin A (ETA/PE)) or plant toxins (ricin and gelonin) chemically conjugated to full-length murine antibodies [35, 36]. Despite showing promising efficacy *in vitro*, their application in the clinical arena was dramatically hindered [37]. Mouse mAbs and non-human toxins gave rise to neutralising antibodies that rendered treatment ineffective [38, 39]. Moreover, the repeated use of high concentrations of such toxins could give rise to side-effects such as vascular leak syndrome and liver injury [40, 41]. Fortunately, molecular techniques allowed for the replacement of murine constant regions of antibodies (F_c_) with human sequences, thus making them “humanized” and less immunogenic [42]. Though a step in the right direction, it is not sufficient; poor penetration of full-length antibodies into a solid tumor mass remained one of the greatest barrier to effective therapy. Using recombinant DNA technology, the 3^rd^ generation of ITs was engineered, consisting of a recombinant antibody derivative genetically fused to bacterial toxins. For example, several PE-based immunotoxins demonstrated promising activity in clinical and pre-clinical studies conducted by the Ira Pastan group [43–45]. While such recombinant ITs (RITs) showed better efficacy, stability and easier distribution in tumor sites, the non-human effector component was not protected from immune rejection. To this end, the next generation of PE-based RITs are now designed with both B and T cell epitopes removed by mutagenesis [46, 47]. Alternative solutions include: chemically modifying proteins with polyethylene glycol (PEGylation) [48], or even replacing existing plant/bacterial toxins with human proteins capable of inducing cell death.

### Human cytolytic fusion proteins: A new generation of human enzymes for targeted cancer therapy

Targeted human cytolytic fusion proteins (hCFPs), a combination of fully human sequences for the antibody, as well as the cytotoxic module, represent a promising future for the treatment of various cancers. To this end, a diverse collection of highly potent human pro-apoptotic proteins has been established. As extensively described by Weidle *et al.* in 2012, these include immunoRNAses, granzyme B (GrB), death-associated protein kinase (DAPk) and death-inducing ligands such as apoptosis-inducing factor (AIF), tumor-necrosis factor (TNF) and TNF-related apoptosis-inducing ligand (TRAIL) [49]. Unlike the other death-inducing ligands, TRAIL, a member of the TNF superfamily of cytokines, has been appealing in the development of biotherapeutic drug candidates that activate TRAIL-receptors (TRAIL-Rs) to induce apoptosis in cancer cells, with little or no effect in normal tissues [50–53]. This tumor-selective treatment approach is independent of both internalization and intracellular routing, and therefore avoids the problem of lysosomal degradation experienced with internalized RITs [54]. However, the winding road leading to the introduction of TRAIL-R agonists in clinical trials, has been marked by several potholes: insufficient agonistic activity of the drug, TRAIL resistance within primary cancer cells and the lack of suitable biomarkers to stratify patients prior to TRAIL-R agonist therapy [50, 55–57].

**Figure 1:**
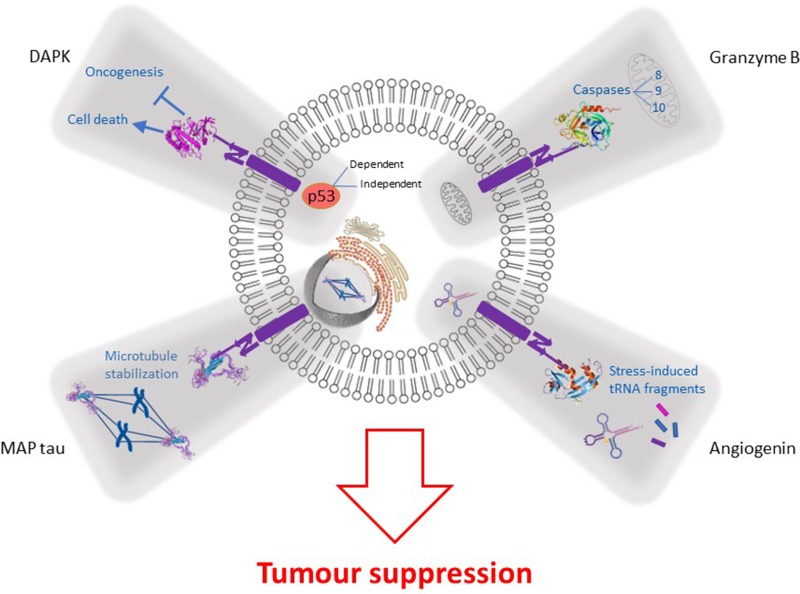
Mechanism of action of targeted human cytolytic fusion proteins (hCFPs) comprising of various effector domains: namely, microtubule-associated protein tau (MAP tau), angiogenin (Ang), granzyme B (GrB) and death-associated protein kinase (DAPk) The success of hCFPs rely broadly on 3 main processes: (1) recognition and binding of the antibody fragment to the target receptor (or upregulated tumor-associated antigen), (2) internalization and (3) delivery of the lethal molecule to the cytosol of the tumor cell. Here, the unique properties of the cancer-killing molecule modulate the activation of various intracellular biochemical reactions that culminate in the apoptosis of the cell: MAP tau induces constant microtubule stabilization, resulting in cell cycle arrest; Ang produces stress-induced tRNA fragments which inhibit protein biosynthesis; the action of GrB activates several caspases which play important roles in programmed cell death; lastly, DAPk mediates p53-dependent/independent apoptosis to suppress tumor growth and metastasis.

In summary, several challenges were associated with cell-death inducing ligands (immunogenicity, toxicity and the lack of clinical benefit in cancer patients [49, 58]), spurring the focus towards the remaining aforementioned human lead enzymes. In order to promote the selective killing of tumor cells, hCFPs must be internalized (presumably by receptor-mediated endocytosis), must be able to escape from the endosomes and eventually be processed for the effective delivery of their cytotoxic cargo into the cytosol of the cell. Once this is achieved, most of these proteins rely on different mechanisms (Figure 1) that all culminate in the induction of apoptosis in diseased cells. Indeed, the strategy behind the design of these hCFPs involve the use of apoptosis as a therapeutic target. This allows for cancerous cells to be removed in a regulated manner, while avoiding the activation of inflammatory reactions, as well as any leakage of cellular content.

Since 2012, continuous innovation has enabled steadily improved performance of hCFPs. For example, revolutionizing computational approaches/simulations have been created to study enzyme-substrate interactions to greater depth, thereby enhancing the enzymatic activity of some human lead candidates (angiogenin and GrB) [59, 60]. As such, this review describes the past and current research conducted in the context of targeted hCFPs encompassing RNAses, GrB, DAPk, as well as the microtubule-associated protein tau (MAP tau), which unlike the others, does not form a classical human enzyme. Additionally, this paper showcases the unique properties and applications of current hCFPs that have propelled them to their current position at the forefront of targeted cancer therapy and innovation.

## MICROTUBULE-ASSOCIATED PROTEIN TAU

### Attacking cancerous cells at their most vulnerable state during mitosis

Before the advent of molecular profiling technologies, it was understood that the accumulation of multiple DNA mutations over time favors carcinogenesis in humans [61]. In most cases, these mutations introduce cell cycle alterations, that confer an unlimited proliferative ability to cells, ultimately resulting in the formation of a tumor. Adopting a holistic approach to preventing the aberrant growth of cells will require one to consider some of the fundamental concepts in cell biology; virtually all cells in the human body rely on a similar molecular machinery and such a system regulates progression through cell division, differentiation and cell death. Considering this approach, many anticancer drugs have been developed to target various important phases in the cell cycle. Most importantly, anti-mitotic drugs have up to now remained the most successful [62]. As a matter of fact, mitosis is a highly coordinated process during which identical copies of the genome are moved to opposite poles of a mitotic spindle, eventually resulting in the formation of two daughter cells [63]. It is subdivided into various phases; namely prophase, pro-metaphase, metaphase, anaphase, telophase and cytokinesis [64]. During metaphase, the chromosomes must be aligned on the equatorial plate by stable microtubule attachment through their kinetochores, before chromatids can be separated and pulled to opposite poles during anaphase [65]. The fidelity of this process is tightly regulated by an independent and evolutionary conserved checkpoint known as the spindle assembly checkpoint (SAC) [66]. Interestingly, current anti-mitotic drugs work by disturbing spindle assembly, activating SAC, causing mitotic arrest and inducing apoptosis [67].

Some of the best known anti-mitotic compounds are the microtubule targeting agents (MTAs) which are broadly divided into microtubule polymerizers (taxanes) and microtubule depolymerizers (vinca alkaloids) [65, 68]. By suppressing microtubule dynamics, these agents affect normal spindle formation and chromosomal orientation, resulting in cell cycle arrest and eventually cell death. Given their potency, such compounds were approved for the treatment of various diseases; including prostate cancer, breast cancer, ovarian cancer, non-small-cell lung carcinoma, and haematological malignancies [68, 69]. However, MTAs lack specificity towards cancer cells and perturb microtubule integrity in important tissues such as the bone marrow and neurons, thereby giving rise to myelosuppression and neuropathy respectively [68]. Furthermore, a major drawback to the use of MTAs is the phenomenon of drug resistance, which can be attributed to mitotic slippage, mutations in tubulin and the overexpression of prosurvival Bcl-2 proteins [70–72]. In view of addressing those needs, a human anti-mitotic protein known as the microtubule-associated protein tau (MAP tau) was identified, showing comparable activities to MTAs and allowing the development of corresponding hCFPs [73].

### The microtubule-associated protein tau as a human cytostatic drug

Being an integral component of the cytoskeleton, microtubules are essential to the existence of eukaryotic cells and are therefore regulated at multiple levels. MAP tau belongs to a family of proteins (known as microtubule-associated proteins or MAPs) which are primarily involved in modulating the stability of microtubules. Studies conducted by Weingarten *et al*. in 1975 revealed co-purification of MAP tau with tubulin, thereby establishing its critical role in microtubule assembly [74, 75]. MAP tau functions by binding to tubulin in a longitudinal fashion, fostering the bridging of tubulin interfaces and hindering the shrinking phase of microtubule dynamics [76]. Thus, the indispensable role of MAP tau warranted its use as an effector protein in antibody-based immunotherapy. Nonetheless, sceptics may point out the strong association between this putative protein and neurodegenerative disorders. For instance, excessive phosphorylation of MAP tau at Ser396, results in the accumulation of extracellular plaques and intraneuronal neurofibrillary tangles ‒ a pathology more commonly referred as Alzheimer's disease (AD) [77–79]. However, MAP tau-based hCFPs were rationally designed to circumvent these health risks through removal of the vital phosphorylation sites of tau (S156 and S204) [73]. Additionally, given the selective nature of antibody fragments towards their target receptors, permeability through the blood-brain barrier (BBB) becomes highly improbable and alleviates the accumulation of MAP tau in the brain.

On that account, the first MAP tau-based hCFP was engineered, bearing specificity towards the human epidermal growth factor receptor (EGFR) while using a constitutively activated MAP tau as a potent cytostatic agent [73]. This involved the design of an expression construct consisting of an anti-EGFR(scFv) genetically fused to MAP tau isoform 3, lacking its vital phosphorylation sites. Interestingly, the choice for isoform 3 is founded on its peculiar nature; it is the lowest molecular weight protein that retains all four highly conserved microtubule binding repeats [73]. Anti-EGFR(scFv)-MAP exhibits specific cytotoxic effect towards cells that are positive for its targeted ligand (Table 1) and no cytotoxicity towards EGFR-negative HEK293 cells. However, the efficacy of this novel effector protein is highly dependent on cell proliferation [73, 80, 81]. This is particularly important since many tumor markers can also be present on physiologically normal cells. Therefore, because rapidly dividing cancer cells represent the target of choice for MAP-tau based hCFPs, any off-target toxicities can be reduced. As a proof-of-concept, apoptosis and tubulin polymerization assays also provided first data to confirm that the cancer-killing mechanism is induced by the constant stoichiometric stabilization of microtubules, resulting in cell cycle arrest and apoptosis. Furthermore, *in vivo* models demonstrated tolerance of anti-EGFR(scFv)-MAP at high doses as compared to the *Pseudomonas* exotoxin A (ETA)-based control [73].

**Table 1:** Cytotoxic activity of human cytolytic proteins with potent human lead enzymes targeting various diseases

Human lead enzyme as effector domain	Construct	Cell lines tested	Disease Model	IC_50_ Values (nM)	References
	αEGFR(scFv)-MAP tau	L3.6pl PC-3 C4-2	Pancreas Carcinoma Prostate Carcinoma Prostate Carcinoma	1000 2500 2800	[72]
	Ki-4(scFv)-MAP tau	L540cy L428 Karpas 299	HL and sALCL HL and sALCL HL and sALCL	53 135 220	[79]
MAP tau	αEpCAM(scFv)-MAP tau	L3.6pl A431 22Rv1 C4-2 SU86.86	Pancreas Carcinoma Epidermoid Carcinoma Prostate Carcinoma Prostate Carcinoma Pancreas Carcinoma	43 67 677 161 333	[80]
	scFv35-MAP tau	FL-OH1 RD	Rhabdomyosarcoma Rhabdomyosarcoma	900 950	[86]
	αCSPG4(scFv)-MAP tau	MDA-MB-231 Hs 578T	TNBC TNBC	219 480	[84]
	H22(scFv)-MAP tau	HL-60 CD64+ leukemic blasts	M1 macrophage-mediated diseases AML/CML	0.04	[82] [83]
	Ang-E6	SF539 MDA-MB-231	Glioma TNBC	15 45	[100]
Angiogenin	αEGFR(scFv)-Ang	A431	SCC	12.5-45	[101]
	MJ7(scFv)-Ang MLT7(dsFv)-Ang	CD22+ tumor cells	Burkitt's Lymphoma	<1000 ~100	[102]
	H22(scFv)-Ang	M1 macrophages	Leukaemia	10 ± 2.7	[103]
	GrB(wt)-H22 (scFv) and GrBR201K-H22(scFv)	Cells from AMML and CMML patients CD64+ HL60	CMML	Not specified 4-7	[156]
Granzyme B	GrB(wt)-ki4(scFv) and GrBR201K-Ki4(scFv)	L428 L540cy	cHL	2.5 1.7	[150]
	GrBR201K-scFv1711	A431 RD	Epidermoid Carcinoma Rhabdomyosarcoma	133.3 21.1	[149]
	GrBR201K-αEpCAM(scFv)	MDA-MB-231 MDA-MB-468 MDA-MB-453	TNBC TNBC TNBC	N/A 221 307	[151]
Death-associated protein kinase	DAPk2Δ73-CD30L	L540 L1236	HL	20 63	[187]
	DK1KD-SGIII	Primary CLL samples	CLL	275-875	[188]

AML: acute myeloid leukemia; AMML: acute myelomonocytic leukemia; HL: Classical Hodgkin's lymphoma; CLL: chronic lymphocytic leukaemia cells; CML: chronic myelomonocytic leukemia; CMML: Chronic myelomonocytic leukemia; CSPG4: Chondroitin sulfate proteoglycan 4; EpCAM: Epithelial cell adhesion molecule; EGFR: Epidermal growth factor receptor; HL: Hodgkin lymphoma; sALCL: systemic anaplastic large cell lymphoma; SCC: squamous cell carcinoma; scFv: single chain fragment variable; TNBC: triple- negative breast cancer.

The use of protein-based effector molecules, such as MAP tau, represents an important turning point in the context of antibody-drug conjugates (ADCs). Through genetic engineering, MAP tau-based hCFPs avoid the need for complex chemistry processes that were previously used to couple cytostatic drugs to their targeting moieties. Compared to their synthetic predecessor, MAP tau-based cytostatic fusion proteins are considered safer since they are produced as an integral whole in a one-step fermentation process using a prokaryotic expression system that represents exciting opportunities for large-scale production [80–82]. Furthermore, several pre-clinical studies promise the application of MAP tau beyond solid tumors; H22(scFv)-MAP tau, which selectively targets CD64+ cells, shows potential for the treatment of atopic dermatitis, rheumatoid arthritis, inflammatory bowel disease and leukemia [83, 84]. More recently, a MAP tau-based fusion protein was created for the elimination of CSPG4-positive triple-negative breast cancers (TNBCs) for which targeted therapeutic treatments are currently lacking [85]. Given the potential clinical value of this fusion protein and the identification of prospective tumor markers, the heterogeneity of such diseases can be approached more effectively via specific patient-tailored therapies.

However, further investigation is necessary to enhance the therapeutic efficacy of MAP tau-based hCFPs. For instance, while the higher IC_50_ values of MAP tau fusion proteins give an indication of their lack of enzymatic activity as compared to ETA, the escape mechanism from the endosomes to the cytosol remains unclear [81]. It has been postulated that once inside endosomes, a pH shift causes a change in protein conformation, resulting in the exposure of potential processing and/or signal sequences [49]. In order to improve the cytotoxic activity of MAP tau fusion proteins, endosomolytic compounds such as chloroquine or wortmannin could be used [86]. Nonetheless, this method would require conjugation, a major complication in the production of heterogeneous ADCs. An improved solution relies on the use of an adapter sequence to facilitate vesicular escape of MAP tau effector molecules to the cytosol of the cell [87]. Moreover, as recently demonstrated by Amoury *et al*., treatment of MDA-MB-231 cells with αCSPG4(scFv)-MAP tau resulted in the induction of the mitochondrial apoptotic pathway, through activation of caspase-9 and endonuclease G translocation to the nucleus [85]. Thus, a better understanding of the apoptosis signaling pathways following mitotic arrest, is necessary to improve the quality of existing MAP tau-based hCFPs for clinical use.

## RNASES

### The potential of human RNases in the development of human cytolytic fusion proteins

ImmunoRNases are a class of targeted therapeutic agents in which the cytolytic component is a native or modified ribonuclease [88, 89]. The intracellular targets of immunoRNases are therefore, by definition, one or more of either mRNA, tRNA or rRNA [90]. Several non-human RNases have been tested as anti-cancer agents, either as stand-alone drugs (untargeted) or in entirely protein-based immunofusions (targeted). Onconase^TM^, a RNase derived from *rana pipiens* and originally known as ranpirnase, had reached Phase III clinical trials as treatment for unresectable mesothelioma [91], but further studies were discouraged due to systemic side-effects experienced by some patients, indicating off-target cytotoxicity. Nevertheless, the apoptosis-inducing potency of this and other RNases, inspired further investigation into combining such enzymes with targeting modalities to increase disease specificity (for a comprehensive review of recently-developed immunoRNases see [92]). The growing emphasis on reducing immunogenicity of protein-based therapeutics led towards identifying human RNases with comparable activities and conditional cytotoxicity for application in recombinant fusion with either human ligands or humanized antibody derivatives [49]. Three human RNases have been reported as potential candidates for the development of hCFPs: human pancreatic RNase (RNase 1) [93–98], eosinophil-derived neurotoxin (RNase 2) [94, 99] and angiogenin (RNase 5) [100–105].

For them to induce apoptosis, RNase-based hCFPs need to be internalized via their target receptor or antigen to gain access to intracellular RNAs. However, pancreatic-type RNases such as RNAse 1 and angiogenin are tightly regulated in both the nuclear and cytosolic compartments by an endogenous ribonuclease inhibitor (RI) [106, 107]. RI-RNase complexes are formed by extremely high affinity interactions and the RNase in question is rendered inactive within this complex [108]. After intracellular delivery, inhibition is the next most limiting problem facing RNase-based hCFPs and reducing susceptibility to inhibition becomes crucial to enhancing *in vivo* cytotoxicity. The generation of RI-resistant variants of angiogenin has been investigated experimentally [109] and more recently refined by means of supercomputing-based simulations of dynamic protein interactions which model the exact regions of interaction between enzyme (angiogenin) and inhibitor (RI) [110].

### Using computational approaches and molecular dynamic simulations to engineer improved angiogenin-based human cytolytic fusion proteins

Angiogenin was the first RNase to be credited with angiogenic activity [111, 112], contributing to later understanding of the wide range of RNase functions. However, angiogenin has been shown to have different effects on cell proliferation depending on the cell's energy state and resource availability. When energy resources are abundant, angiogenin enters the nucleus via a M^30^-R-R-R-G^34^ nuclear translocation signal (NTS) [113, 114] and binds to a CT-repeat angiogenin binding element (ABE), promoting ribosome synthesis, ribosomal RNA transcription and maturation (specifically processing of 18S and 28S rRNA) [112, 115] and thereby driving cell proliferation in several contexts, including contributing to tumor angiogenesis [112]. Under starvation conditions, angiogenin remains in the cytoplasm where it cleaves tRNA, producing stress-induced tRNA fragments (stRNAs) which act as signaling factors to inhibit protein biosynthesis and eventually result in apoptotic cell death due to abrogated biomass production [116, 117]. It is therefore in the best interest of a thriving malignant cell to prioritize inactivation of extra-nuclear angiogenin with high cytosolic RI levels.

Cytosolic RI binds angiogenin in a 1:1 stoichiometry with the highest affinity of any RI-RNase interaction. Since only a slight excess of RI is required to inhibit angiogenin activity in any given cellular compartment, it is possible that the concentration of RI present in said compartment, and not the present angiogenin concentration, is responsible for regulating the compartment-specific activity of angiogenin. Targeted delivery of even a few molecules of RI-resistant angiogenin in the form of hCFPs may thus exert sufficient tRNA hydrolysis to induce apoptosis even in the presence of inhibitor.

Having identified the angiogenin residues residing in the regions of interaction with RI, the authors replaced Gly^85^ and Gly^86^ with arginine residues (G85R/G86R) with the aim of introducing steric and electrostatic hindrance to the formation of the Ang-RI complex. The resulting reduction of enzyme/inhibitor affinity has been shown to enhance cytotoxicity more effectively than attempts to enhance enzymatic activity or substrate affinity [103–105].

Altering enzyme structure based on simulation of dynamic protein interactions is not only restricted to inhibitor binding affinity but can also be extended to substrate interaction and catalytic activity. Residues involved in substrate binding and catalysis are somewhat conserved between angiogenin homologues and yet angiogenin exhibits the weakest substrate affinity within the pancreatic RNase superfamily. Angiogenin residues Gln^12^, Thr^44^, Asn^68^, Glu^108^ & Ser^118^ (respectively corresponding with Gln^11^, Thr^45^, Asn^71^, Glu^111^ & Ser^123^ in RNase A) are believed to mediate enzyme-substrate interaction, while His^13^, Lys^40^ and His^114^ (respectively corresponding with His^12^, Lys^41^ and His^119^ in RNase A) form part of the active center for ribonucleolytic activity [112, 118, 119]. One marked difference between the angiogenin and RNase A involves the presence of the Gln^117^ residue within the putative substrate-binding region of angiogenin. This large and reputably obstructive residue corresponds with an Ala in the binding region of RNase A and appears to result in comparatively weak RNA substrate affinity [120]. This has been verified by the observation that substitution of Gln^117^ with a less obstructive residue (Ala or Gly) appears to increase angiogenin enzymatic activity [104].

The design of enhanced enzyme variants does appear to be the next step in the improvement of RNase-based hCFP development for targeted therapeutic applications.

## GRANZYME B

### Granzyme B: Its importance and mechanism of action

Cytotoxic lymphocytes in the form of natural killer cells and cytotoxic T lymphocytes (CTL) play a pivotal role in defending the body against infections or the formation of malignant cells [121]. The defense is catalytically activated through the receptor mediated Fas-Fas ligand pathway as well as granule exocytosis pathway. Granule exocytosis is facilitated through the synergistic relationship between pore forming perforins and pro-apoptotic serine protease granzyme family [121–123]. The importance of this relationship has been documented in multiple studies including Greenberg *et al.* in the early 1800 who initially showed that both perforins and granzyme B (GrB) were required to induce DNA fragmentation in targeted cells [124, 125].

The 32 kDa GrB serine protease has been shown to be the most lethal granzyme in several studies [124, 126–130]. Due to its potent nature, GrB is expressed in cytotoxic lymphocytes as an inactive prepro-enzyme harboring an N-terminal signaling peptide sequence [131]. Upon recognition of a viral threat or formation of a malignant cell, the signaling peptide is processed in the endoplasmic reticulum (ER) leading to the glycosylation of GrB with two mannose phosphate groups [132]. This glycosylation marks GrB for packaging into secretory vesicles within the cytotoxic lymphocytes. In the secretory vesicle, GrB is stored in a complex consisting of serglycin and perforin. Activation of GrB is facilitated through the lysosomal dipeptidyl-peptidase I (DPPI) removal of dipeptide Gly-Glu from its N-terminus. This leads to allosteric changes in the activation domain rendering the enzyme catalytically drawn to the cleavage of aspartic-containing residue [133, 134].

GrB is directed from cytotoxic lymphocytes vesicles to the targeted cell through a calcium dependent synaptic portal. The mechanism by which GrB crosses the target cell membrane into the target cell cytosol depends strongly on the presence of perforins [135, 136]. Perforins form pores on the targeted cell lipid membrane and disrupts endosomal trafficking. The cytosol contains multiple GrB specific substrates with the aspartate or glutamate at the P1 site of a tetrapeptide motif therefore enabling it to cleave substrates directly or indirectly [137, 138]. In the caspase independent pathway, GrB cleaves pro-caspase 8 leading to its dimerization and activation, and subsequent activation of the mitochondrial pathway. GrB can also directly cleave the BH3 interacting domain death antagonist (BID) leading to the formation of truncated BID (t-BID) which directly activates the mitochondrial pathway by translocating into the mitochondria and activating pro-apoptotic Bcl-2 family BAX and BAK. The activation of these proteins leads to mitochondrial outer membrane permeabilization (MOMP) and release of cytochrome C, Smac/DIABLO and OMI/HTRA2 which promote the blocking of inhibitor of apoptosis protein (IAP). Cytochrome C release in the presence of ATP results in the binding of the apoptotic protease activating factor (APAF-1) and the subsequent formation of the apoptosome which activates procaspase 9 leading to subsequent activation of the precursor apoptotic caspase 3 and 7. Caspase dependent activation of procaspase 3 and 7 directly by GrB culminates in programmed cell death leading to the DNA fragmentation, cell shrinkage, formation of apoptotic bodies and chromatin condensation [139–142].

### Granzyme-B based targeted human cytolytic fusion proteins

The adoption of GrB as an effector enzyme in the design of hCFPs, is based on its strong cytotoxic activity, its diverse apoptosis inducing mechanism, its human origin and its controlled activation. Once processed in the cell, the induction of apoptosis using GrB-based hCFPs occurs directly through the caspase dependent pathway or indirectly through the mitochondrial pathway/caspase independent pathway. However, the independence of GrB-derived hCFPs from the presence of perforins in eliciting an efficient immune response requires the structural design of the recombinant fusion protein to be significantly changed [132]. As discussed previously, activation of GrB requires correct processing in the ER and the presence of a free N-terminus. Multiple studies have previously demonstrated the successful expression of GrB and GrB-based hCFPs in bacterial, mammalian, yeast and insect cells [142–144]. For instance, expression of GrB in yeast *Pichia Pastoris* necessitated the presence of *Sacchromycces cerevisiae* alpha mating secretion signaling peptide to enable processing in the golgi apparatus by Kex2 protease. Additionally, insertion of an enterokinase cleavage site upstream of a mature polypeptide enables *in vitro* processing. The later strategy has been largely adopted for GrB hCFP expression [145, 146]. In order to avoid cytotoxic destruction of the GrB expressing cell, the GrB-based hCFP is secreted as an inactive zymogen with a pre-peptide sequence followed by an enterokinase sequence guarding the N-terminal side. Using this strategy, GrB is activated only upon processing of the enterokinase cleavage site.

Following this systematic activation of GrB using enterokinase cleaving enzymes, GrB has been fused to a plethora of antibody fragments and ligands to target CD64, HER2, gp40, CD30, EpCAM and EGFR [144, 147–150]. The apoptosis inducing effector function of GrB has been shown to induce cytotoxicity in the nanomolar range similar to that induced by the ETA toxin. However, the cytotoxicity is often not as significant due to endosomal escape. In order to achieve the picomolar ranges, cells need to be treated with endosomolytic agents such as chloroquine [147, 149]. This enables the disruption of endosomal functionality resulting in the activation of the GrB-based hCFP in the cytosol.

### Counteracting serpin B9 inhibition using supercomputing platforms

It is now well understood that the highly toxic nature of GrB is controlled at both the translational and post-translational level. The latter control mechanism involves the presence of GrB natural inhibitor serpin B9 which is present in the cytosol of cytotoxic lymphocytes to prevent self-inflicted apoptotic induction caused by granule leakage and the misdirected activity of GrB [151]. Serpin B9 has also been found to be upregulated in tumor cells which therefore enables tumor cells to evade GrB-induced apoptosis. Serpin B9 blocks GrB activity through a stoichiometric reaction resulting in the deactivation of both enzyme and inhibitor. In this reaction the serpin B9 residue in the variable reactive center loop (RCL) P1 position is a glutamate which is highly specific for protease cleavage [152, 153]. Therefore, recognition of the cleavage site via GrB leads to inactivation. To counter the effects of this inhibition, hCFPs conferring resistance to serpin B9 inhibition have been successfully designed. Supercomputing platforms enabling the mapping of amino acids which play a role in serpin B9 reactive center loop (RCL) and serine protease binding during an inhibitory reaction have been instrumental in this process. The ROBETTA server identified two interface proteins at position R28 and R201 which were manipulated using site-directed mutagenesis such that arginine was replaced with alanine (neutral charge (A)), glutamate (Opposite charge (E)) and lysine (same charge (K)) [153, 154]. Seven PI-9 resistant variants were generated: R28A, R28K, R28E, R201A, R201K, R201E and the double mutant R28A-R201A. *In silico* modelling using Baker's computer-aided simulation modelling (CASM) procedure [60] followed by molecular dynamics simulations of PI-9 complexes with wild-type and mutated GrB in aqueous solution showed that GrB mutation at position 28 conferring the lysine mutation and GrB mutation at position 201 conferring the alanine mutation (GrBR28K, GrBR201A and GrBR201K), remained catalytically active and inferred resistance. However, after *in vitro* assays using the GrB mutated variants to cleave the synthetic substrate AC-IEDT-*p*NA (which mimics the cleavage site of the GrB substrate procaspase-3), it was found that GrB hCFPs conferring the R201K modification showed the most efficiency with activity surpassing the wild type. Furthermore, the *in vivo*, *ex vivo* and *in vitro* successful application of GrB 201K-conferring fusion proteins were demonstrated in the design of hCFPs comprising wild-type GrB or GrBR201K fused to the antibody Ki4(scFv), which targets CD30 overexpressed on classical Hodgkin's lymphoma cells. In this study, L428 and PI-9-negative L540cy Hodgkin's lymphoma cell lines expressing PI-9 and a mouse subcutaneous tumor model based on L428 cells were shown to completely abolish the activity of wild-type GrB, whilst having no effect on the mutated variant [149]. Since then, GrB 201K fusion proteins targeting EpCAM in TNBC, H22 in Chronic myelomonocytic leukemia and EGFR in Epidermis cancer cells have been reported (Table 1). An additional detailed report of Granzyme B based human cytolytic fusion proteins designed with wild type Granzyme B activation can be found in Hehmann-Titt *et al.* (2013) [151].

### Further improvements in the activity of Granzyme B

The specificity of hCFPs is not only governed by the targeting scFv but also by the effector enzyme. To this regard, GrB contains a high isoelectric point (pI) which creates a net positive charge on the protein surface facilitating the electrostatic interactions with the negatively-charged heparan sulphate proteoglycans found on the surface of almost all cells [155]. This reduces the therapeutic efficacy of the enzyme because it becomes trapped on non-target tissues. To prevent unspecific binding, GrB-based hCFPs are designed such that the antibody component has a low pI and the combined charge is lower, but the cytotoxic efficacy is unaffected [155]. The heparin-binding motifs RKAKRTRA and KKTMKR on mature GrB confer a positive surface charge and thereby facilitate the interaction with glucosaminoglycans on the cell surface [156]. The basic amino acid residues involved in heparan sulfate binding were substituted for alanine to reduce cell binding and endocytosis [156]. The substitution of cationic sequences has no impact on the enzymatic and pro-apoptotic activity of GrB. This GrB hCFP variant (GrBcs) showed reduced nonspecific binding to EGFR-negative MDA-MB-453 cells as well as optimal activity in the presence of EGFR-positive and EGFR-negative cells [152, 157].

Ongoing developments are underway to continuously improve the therapeutic potential and specificity of GrB-based hCFPs. The growing application of GrB in immunotherapy is being realized. In the context of hCFPs phenomenal strides have been achieved in addressing the resistance to serpin B9 and tailoring the specificity. However, there is still room for improvement in increasing the cytotoxic potential as comparable to that achieved by bacterial-derived immunotoxins. In the future, we envisage a GrB-based fusion protein that is highly specific, demonstrates potent and controlled cytotoxic activity, is efficiently routed in the endosomes and targets multiple receptors.

## DEATH-ASSOCIATED PROTEIN KINASE

### The death-associated protein kinase as a potential target in therapeutic interventions

Apoptosis is a regulatory mechanism which allows multicellular organisms to tightly control cell numbers, tissue size and to protect themselves from rogue cells that threaten homeostasis [158]. Unfortunately, several lines of evidence suggest the acquisition of certain hallmarks that endow an unlimited proliferative potential to cells [159], to counteract apoptosis and promote tumorigenesis. For instance, the lack of epigenetic control is heavily implicated in the development and progression of cancer [160]. Several genes that play important roles in tumor suppression, DNA repair and metastasis are disproportionately methylated in tumor cells, resulting in their transcriptional repression [161]. One such gene is the death-associated protein kinase (DAPk), whose promoter regions are known to be hypermethylated in a wide range of tumors [162, 163].

DAPk (also known as DAPk1) is the prototypic member of a family of five Ca^2+^/Calmodulin (CaM)-dependent serine/threonine kinases (DAPk1, DAPk2, ZIPk, DRAK-1 and DRAK-2) that are known to suppress tumor growth and metastasis, by mediating a wide range of cellular processes, including p53-dependent or independent apoptosis and autophagy [164–166]. DAPk1 was first discovered in the mid-1990s in an elegant study designed to identify genes necessary for interferon-γ (IFN-γ)-induced death in HeLa cells [167]. Since then, this 160 kDa kinase has been shown to regulate diverse biological signals; membrane blebbing, autophagy, growth factor-induced survival and cancer development, amongst others [168]. Among the DAPk family of proteins, DAPk2 and ZIPk are known to share a high degree of similarity to DAPk1's catalytic domain, accounting for 83% and 80% identity at amino acid level respectively, whereas DRAK-1 and DRAK-2 share only 50% homology with DAPk1 [165, 169–171]. Most importantly, the tight conservation of this catalytic domain correlates with several shared properties, including common substrates and similar functional effects [169]. To illustrate this point, DAPk1, DAPk2 and ZIPk are proposed to form a unique kinase hierarchy that culminates in the phosphorylation of ZIPk and subsequent activation of death-promoting signals [172]. Detailed insights into the characteristic structural features and mechanism of action of the DAPk family has been well-documented [165, 166, 173], and activated DAPk proteins are reported to initiate a variety of death pathways depending on the cellular context [174].

Nonetheless, an understanding of the underlying molecular mechanisms that play a major role in DAPk1's transcriptional regulation constitutes a prerequisite to the development of novel therapeutics against cancer and inflammation-associated diseases [175]. Strikingly enough, a detailed analysis of the mRNA and protein expression profile of DAPk1 in various tumor cell lines, including bladder, breast and renal carcinomas, led to the discovery of loss of DAPk1 expression [176]. In most cases, this strange phenomenon was not caused by a deletion or rearrangement of the DAPk1 gene, but due to epigenetic silencing [174]. As such, hypermethylation of the 5′ CpG island of DAPk1's promoter region is common in many cancers and occurs at varying levels [177, 178], although DAPk1 expression loss can also be a result of homozygous deletion [179]. Additionally, a study conducted by Chen *et al*. in 2012 demonstrated the role of microRNAs in inhibiting DAPk1 translation, thus promoting metastasis in colorectal cancer [180]. Given the functional role and varied DAPk1 status in disease, manipulation of DAPk1 expression or activity may represent a promising approach for therapeutic interventions [181].

On the basis that DNA methylation is a reversible process, drugs capable of demethylating DNA and re-activating silenced genes (5-aza-2′-deoxycytidine and 5-azacytidine), were developed and tested in patients [169]. One such agent, most commonly known as Decitabine (5-aza-2′-deoxycytidine), was shown to reactivate DAPk1 expression, as well as the cell's apoptotic sensitivity to IFN-γ [177]. Similarly, a proof-of-concept study demonstrated that Decitabine also restored down-regulated DAPk2 tumor suppressor activity in Hodgkin's lymphoma cells [182]. Unfortunately, despite their promising value as cancer therapeutics, such drugs were limited by their chemical instability, inability to target specific genes and unsuitability as an orally administered drug [169]. In light of the above arguments, unravelling the cellular and molecular complexities of the DAPk family has provided the necessary milestones for the establishment of recombinant immunokinase fusion proteins that can restore apoptosis in a targeted and specific manner.

### DAPk-based fusion proteins

Of paramount importance is the role of autophosphorylation, as a unique regulatory mechanism of DAPk1 and DAPk2, which is relieved through the binding of Ca^2+^-activated Calmodulin to the CaM regulatory domain [183, 184]. Therefore, it was postulated that the deletion of this domain would result in mutants (DAPk1ΔCaM and DAPk2Δ73) that are constitutively activated to stimulate apoptosis via a variety of pro-apoptotic and/or autophagic signals [185]. Using this approach, the first DAPk2-based fusion protein, a human DAPk2Δ73 genetically fused to the extracellular domain of human CD30L, was developed for the treatment of Hodgkin's lymphoma [186]. The authors showed that a single injection of DAPk2Δ73-CD30L prevented tumor development in mice xenografts, while raising awareness for the need to thoroughly assess DAPk2 expression in primary Hodgkin lymphomas.

More recently, a novel CD22-targeting fusion protein containing a constitutive DAPk1 mutant (DK1KD-SGIII) showed effective binding, internalization and cell death induction on malignant B cells [187]. The IC_50_ values on primary chronic lymphocytic leukaemia (CLL) samples varied from 275–875 nM, without any effect on CD22-negative cells from healthy donors or CLL-patients (Table 1). Moreover, a deeper look into the mechanism of action of DR1KD-SGIII revealed not only classical caspase- and PARP-mediated apoptosis, but also expression of the autophagic marker LC3B in CLL cells [187]. Similarly, this paper also highlights the manufacturing feasibility of such fusion proteins with high-yield, preserved binding and robust catalytic activity.

DAPk-based fusion proteins exhibit two major advantages over the other hCFPs described in this review. Firstly, most tumor-associated antigens that are upregulated in various cancers, are also present on normal cells, which can give rise to off-target effects. As shown by Tur *et al.*, cells that showed normal expression of DAPk2 were not affected by this therapeutic approach [186]. Secondly, through the use of a reconstituted catalytic tumor suppressor, DAPk-based fusion proteins are able to bypass resistance mechanisms, unlike Ang and GrB which are limited by the upregulation of their endogenous inhibitors in target cells.

## CONCLUSIONS

Several biologically useful proteins originating from plants and bacterial species (Ricin, Gelonin, ETA and Diphtheria) form an attractive source of biopharmaceuticals [35, 36, 188]. Despite their high potency and efficacy, they are recognized as foreign by the immune system, which considerably limits the number of treatment cycles that can be administered to patients [49]. As such, the incidence of immunogenicity after a single cycle of immunotoxin treatment ranges from 50–100% for solid tumors and 0–40% for hematologic tumors [34]. For instance, while various ETA-based immunotoxins have been developed (see [34] for more details), only patients with Hairy Cell Leukemia, whose immune systems are compromised by the cancer, are able achieve complete regressions and prolonged life [45, 189]. However, in patients with normal immunological responses, the presence of neutralizing antibodies reduces the amount of biologically active immunotoxin, thereby affecting its efficacy.

While the work of Pastan and colleagues resulted in successful de-immunization of ETA-containing immunotoxins [59, 190], another potential solution to the problem of immunogenicity, involves substituting existing plant/bacterial toxins with human pro-apoptotic proteins. As extensively described in this review, with a growing cadre of human effector candidates such as MAP tau, Ang, GrB and DAPk, various apoptotic pathways can now be exploited to selectively induce tumor cell death. With the increasing popularity of supercomputational approaches in studying enzyme-substrate interactions, the activity of current human toxins with endogenous inhibitors (Ang and GrB) can be further optimized [59, 60]. However, the identification of novel human translocation domains might be indispensable in the development next-generation hCFPs with improved efficacy and cytosolic toxin-release. Nonetheless, hCFPs represent a promising tool in the armamentarium of therapeutics available for treating a wide range of malignancies.
